# Vegetarianism and colorectal cancer risk in a low-selenium environment: effect modification by selenium status? A possible factor contributing to the null results in British vegetarians

**DOI:** 10.1007/s00394-016-1364-0

**Published:** 2017-02-13

**Authors:** Jakub G. Sobiecki

**Affiliations:** 1 Cancer Epidemiology Unit, Nuffield Department of Population Health, Richard Doll Building, University of Oxford, Roosevelt Drive, Oxford, OX3 7LF UK; 2 Department of Epidemiology and Biostatistics, School of Public Health, Imperial College London, St Mary’s Campus, London, W2 1PG UK; 3 Department of Paediatrics, Nutrition and Metabolic Disorders, Children’s Memorial Health Institute, Al. Dzieci Polskich 20, Warsaw, 04-730 Poland

**Keywords:** Vegetarian, Selenium, Colorectal cancer, United Kingdom

## Abstract

**Background:**

Despite the consistent findings of lower total cancer incidence in vegetarians than in meat-eaters in the UK, the results of studies of colorectal cancer (CRC) risk in British vegetarians have largely been null. This was in contrast to the hypothesis of a decreased risk of CRC in this population due to null intake of red and processed meats and increased intake of fibre. Although the data are inconsistent, it has been suggested that selenium (Se) status may influence CRC risk.

**Methods:**

A literature review was performed of studies on CRC risk in vegetarians, Se intakes and status in vegetarians, and changes of Se intakes and status in the UK throughout the follow-up periods of studies on CRC risk in British vegetarians.

**Results:**

Vegetarians in the UK and other low-Se areas were found to have low Se intakes and status compared to non-vegetarians. There was some evidence of a reverse J-shaped curve of Se intakes and status in the UK throughout the last three decades. These presumed patterns were followed by the changes in CRC mortality or incidence in British vegetarians during this period.

**Conclusions:**

Available data on Se intake and status in British vegetarians, as well as the relationship between their secular changes in the UK and changes in CRC risk in this dietary group, are compatible with the hypothesis that low Se status may contribute to the largely null results of studies of CRC risk in vegetarians in the UK.

## Introduction

According to the World Cancer Research Fund (WCRF), there is convincing evidence that processed (i.e. preserved by smoking, curing or salting, or addition of chemical preservatives) and red meats, as well as foods high in fibre, are causative risk factors for colorectal cancer (CRC), with the former increasing and the latter decreasing its risk [1]. The recent evaluation of the carcinogenicity of these foods by the International Agency for Research on Cancer has corroborated the findings of the WCRF by classifying processed meats as Group 1 carcinogen (“carcinogenic to humans”) and red meat as Group 2A carcinogen (“probably carcinogenic to humans”), based chiefly on the evidence for CRC [2]. It is estimated that minimizing the intake of processed and red meats (to nil intake) could prevent 21% of cases of CRC in the UK, while eating a high-fibre (≥23 g fibre per day) diet could prevent 12% of cases [3].

In light of these findings, it seems reasonable to propose a vegetarian diet emphasizing minimally processed plant-based foods as a model for risk reduction of CRC. Indeed, a 46% reduction of 6 year incident colon cancer risk (rectal cancer and CRC were not reported), but not CRC mortality [4], has been observed in American vegetarians within the Adventist Health Study 1, compared to otherwise similar members of the Adventist Church [5]. Also, the authors found a positive association of total, red and white meat intakes with colon cancer risk; a negative association between legume intake and this risk; and evidence of interaction between high red meat intake, low legume intake, and high body mass in increasing the risk [5].

In the largest cohort study which purposely recruited a high proportion of vegetarians—the ongoing Adventist Health Study 2—after a mean follow-up of 7.3 years, the relative hazard of incident CRC was 22% lower in all diet groups with reduced or nil meat intake (semivegetarians, pesco-vegetarians, vegetarians and vegans) combined than in non-vegetarians, and the observed risk reduction in colon, but not rectal cancer in all types of vegetarians combined was close to statistical significance (HR 0.81; 95% CI: 0.65–1.00, *p* = 0.053) [6]. In analyses of separate vegetarian diet groups, only pesco-vegetarians had a significantly reduced risk of CRC incidence (HR 0.57; 95% CI: 0.40–0.82).

The results of studies in British vegetarians—historically the only cohorts other than the Adventist cohorts with sufficient sample sizes to detect differences in CRC rates between vegetarians and non-vegetarians—have largely been null, both in regard to CRC mortality [7, 8] and CRC incidence [9–11].

## CRC risk in British vegetarians—an overview of the published findings

Six reports on CRC risk in British vegetarians were published thus far from three studies: Health Food Shoppers Study (HFSS) [7], the Oxford Vegetarian Study (OVS) [8], the Oxford arm of the European Prospective Investigation into Cancer (EPIC-Oxford) study [9, 12] and combined analyses from the EPIC-Oxford and the OVS [10, 11]. The details on study design and recruitment of subjects have been described elsewhere [13–15].

Briefly, the HFSS and the OVS recruitment took place in the 1970s and early 1980s, respectively, with follow-up until the late 1990s. Both cohorts comprised approximately 11,000 subjects each, with health-conscious non-vegetarians and a large proportion of vegetarians (ca. 40%). In the ongoing EPIC-Oxford study, a cohort of 65,429 subjects was recruited between 1993 and 1999, comprising ca. 30% of vegetarians, 15% of fish-eaters (pesco-vegetarians) and 4% of vegans. The remaining meat-eaters included ca. 80% of health-conscious individuals, with the rest being recruited from the general population. In all three studies, follow-up was by record linkage with the National Health Service Central Register.

In both the HFSS and the OVS, the CRC mortality risk was insignificantly lower in vegetarians than in non-vegetarians at 0.85 (95% CI: 0.52–1.39) [7] and 0.85 (95% CI: 0.55–1.32) [8], respectively, after a mean follow-up of 21 and 17 years, respectively.

In the EPIC-Oxford study, the relative rate of CRC incidence was in fact higher in vegetarians than in meat-eaters after a mean follow-up of 10.7 years (RR 1.39; 95% CI: 1.01–1.91), despite the total cancer incidence being borderline significantly lower (*P* = 0.052) by 11% in vegetarians [9]. Also, the standardized incidence ratio (SIR) of 101 (95% CI: 79–128) suggested that vegetarians had incidence rate of CRC similar to average British citizens, while non-vegetarians (meat-eaters and fish-eaters combined) had significantly lower CRC SIR (84; 95% CI: 73, 95). In line with these findings, the standardized mortality ratio (SMR) for CRC in EPIC-Oxford after the same mean follow-up was significantly lower in non-vegetarians than in the general UK population (SMR 67; 95% CI: 54–82), but not in vegetarians (81; 95% CI: 55–115) [12]. This was despite the SIRs and SMRs for all malignant neoplasms being significantly and substantially lower in both non-vegetarians and vegetarians than in the general population—all at approximately 70 [9, 12].

Subsequent reports of combined analyses of incident CRC risk from EPIC-Oxford and OVS were null after 12.2 years average follow-up (RR 1.12; 95% CI: 0.87–1.44) [10] and 14.9 years (RR 1.03; 95% CI: 0.84–1.28) [11]. Again, this was despite the total cancer incidence in vegetarians being significantly lower than in meat-eaters by 12% in both publications. In the latter analysis, a decreased risk of CRC was observed in fish-eaters compared to meat-eaters (RR 0.66; 95% CI: 0.48–0.92) [11].

It should be emphasized that chance may be one plausible, at least partial explanation for the rather surprising findings of increased CRC risk in vegetarians, given the null results in all other publications from this population and the lower end of the 95% confidence interval being close to 1. It is important not to make a case against the established, negative health effects of processed and red meats or dietary patterns including these foods by selectively citing the results of increased CRC risk in British vegetarians, as it has been done in the literature in relation to CRC [16], as well as coronary heart disease and type 2 diabetes [17].

## Possible causes of differences in CRC risk between American and British vegetarians

Fraser has proposed that the characteristics of the dietary pattern of the EPIC-Oxford cohort may be important to the inconsistency between the result of American and British studies of CRC risk in vegetarians [18]. Other than null or episodic intake of meat in vegetarians and increased intake of fruit and vegetables, their dietary pattern is similar to that of the UK population; however, none of its characteristics [19] seem to be particularly relevant to CRC risk, at least in light of the current knowledge [1].

Also, it should be noted that the intake of meat in non-vegetarians in the EPIC-Oxford study is relatively low at a median of 65 g/day in men and 54 g/day in women [9]. For reference, the mean national intakes in British adults are estimated to be 130 g in men and 89 g in women [20]. It appears that the differences in nutrient intakes between vegetarians and non-vegetarians are less pronounced in the EPIC-Oxford cohort, compared to those observed in the Adventist Health Study-2 [6, 21, 22]. However, it may be at least in part due to the differences in the food frequency questionnaires (FFQ) used in the studies, with the AHS2 FFQ (unlike the EPIC-Oxford FFQ), allowing participants to choose different multiples of the standard portion size. Due to the lower energy density of staple plant foods, such as whole grains and legumes, vegetarians may be more likely to consume larger portions than meat-eaters; therefore, a single serving size FFQ may underestimate their intakes of these foods and thus most nutrients.

When investigating the possible dietary factors influencing the CRC risk in British vegetarians, it is important to note that in contrast to the USA, the UK (as well as most of Europe) is a low-selenium (Se) area [23]. To illustrate the magnitude of the difference, mean daily Se intakes are approximately 40 μg per day in Europe, and 93 μg in American women and 134 μg in American men [24]. For reference, the daily level of intake currently recommended in the UK for individuals is 1 μg of Se per kg of body weight, which in practical terms translates to recommended intakes of 60 and 75 μg/day for females and males, respectively [25].

Given that Se status has been implicated in cancer risk in humans [24, 26], and that vegetarians in low-Se areas have lower Se intake and status than non-vegetarians (Table 1), it seems prudent to explore the speculative hypothesis that it may have been involved in the CRC risk in the studies of British vegetarians.

**Table 1:** Se status and dietary Se content in duplicate diet studies in European vegetarians

Author, year	Country	Non-vegetarians	Vegetarians and vegans			Comments
			Vegetarians	Vegans		
Toenail selenium content (ng/g)						
Judd et al., 1997 [63]	UK	685	541		14% of vegetarians reported occasional consumption of tuna and had mean concentrations of 644 ng/g	
			591	506		
Serum or plasma selenium (ng/ml)						
Kadrabová et al., 1995 [65]	Slovakia	58	48	–	Erythrocyte Se, plasma and erythrocyte GPx activities also lower in vegetarians	
Kováciková et al., 1998 [66]	Slovakia	*Males*			“The majority of vegetarians were actually semivegetarians.” Erythrocyte Se, plasma and erythrocyte GPx activities also lower in vegetarians	
		57	50	–		
		*Females*				
		57	49	–		
Krajcovicová-Kudlácková et al. 1995 [73]	Slovakia	*Males*			Methodological uncertainties—see section “Are vegetarians in the UK at increased risk of low selenium status?”	
		52	65	–		
		*Females*				
		58	62	–		
Nagyova et al., 1995 [67, 68]	Slovakia	Significantly lower plasma Se in vegetarians			Full text or the abstract of the article no longer available (personal communication with Ginter). Plasma GSH and GPx also lower in vegetarians	
Krajcovicová-Kudlácková et al., 1995 [74]	Slovakia	Significantly higher Se status in adolescent vegetarians.			Full text or the abstract of the article not available. Non-Medline indexed journal Cited in [61] without specifying Se parameters	
Hoeflich et al., 2010 [69]	Germany	93	74		Values read from figure. Serum SePP, but not GPx3 activity, lower in vegetarians and vegans combined. NS differences in Se status between vegetarians and vegans	
			76	71		
Akesson et al., 1985 [70]	Sweden	82	63	74	NS difference between vegans and non-vegetarians	
Srikumar et al., 1992 [71]	Sweden	76	55	–	1-year trial assessing trace element status after adopting a vegetarian diet (non-vegetarian—baseline Se); values read from figure.	
Elorinne et al., 2016 [72]	Finland	118	–	87	Se added to fertilizers in Finland. Low numbers of subjects (22 vegans, 19 non-vegetarians)	
Selenium diet content (μg/day)						
MAFF, 2000 [55]	UK	39	–	28	7-day diet duplicates in vegetarians. Value for non-vegetarians taken from Total Diet Study 1999 estimate [56]. NT	
Roekens et al., 1986 [59]	Belgium	50	13		24 h diet duplicates. Mean daily Se content of macrobiotic diets was 34 μg. Value for non-vegetarians read from figure. NT	
Abdulla et al., 1981 [60]	Sweden	31	–	7.8	4-day diet duplicates in vegans and 7-day diet duplicates in (elderly) non-vegetarians	
Abdulla et al., 1984 [62]	Sweden	–	68	–	4-day diet duplicates. High content likely due to a large proportion of foods being imported from Se-rich areas. NT	

All row comparisons of vegetarians and/or vegans with non-vegetarians significantly different at *P* value < 0.05, unless otherwise noted in the Comments column

*Se* selenium, *GPx* glutathione peroxidase, *GSH* glutathione, *SePP* Se-transport protein selenoprotein P, *NS* non-significant, *NT* no statistical test performed, *MAFF* Ministry of Agriculture, Fisheries and Food

Interestingly, there is scant evidence to support the notion that a vegetarian diet in the UK may upregulate some mechanisms involved in the development of CRC. Joosen et al. [27] carried out two small, randomized crossover studies comparing the short-term effect of a vegetarian diet with omnivorous diets containing either red or processed (nitrite-preserved) meats on endogenous nitrosation and DNA damage. In both of these comparisons, there were significantly more faecal water-induced DNA strand brakes in Caco_2_ cells in the vegetarian diet groups, as measured by the comet assay. The measurement of faecal water genotoxicity suffers from considerable technical difficulties [28], and hence the described results should be interpreted with due caution. That being said, it is reasonable to expect at least one aspect of the vegetarian diet in the UK to negatively influence the CRC risk, given that its significantly decreased risk has not been observed in British vegetarians, despite no intake of red and processed meats and higher intake of fibre than that of meat-eaters [1, 15], as well as a higher frequency of bowel movements [29].

## Selenium and cancer in humans

Se is an essential nutrient, which is utilized for the expression of 25 different selenoprotein-encoding genes. The majority—and possibly all—of the selenoproteins are involved in the regulation of the redox status [30]. Some of them, namely glutathione peroxidases (GPxs), 15 kDa selenoprotein, Se-transport protein selenoprotein P (SePP) and thioredoxin reductases, have been implicated in tumourigenesis or cancer spread [26]. Although the biology of Se is highly suggestive of a protective effect against cancer, which indeed has been shown in various animal models [29], the findings from studies in humans have been conflicting. It is currently unclear whether or not Se intake has a direct influence on cancer risk in humans, and more specifically CRC risk in the context of the current review.

The WCRF Expert Panel has judged foods containing Se to be a probable factor decreasing the risk of prostate cancer, and that there is limited-suggestive evidence for the same effect of this food group in the context of CRC, lung and stomach cancers [31]. In the subsequent WCRF Continuous Update Project (CUP) report on prostate cancer, judgement was made in regard to “low blood levels of Se” rather than the intake of foods from this food group, and the level of evidence was downgraded to limited-suggestive [32]. In the WCRF CUP report on CRC, the level of evidence for selenium’s effect on CRC risk was also downgraded (to limited-no conclusion), both in regard to foods containing Se and supplementary Se [1].

A recent Scientific Advisory Committee on Nutrition position statement on selenium and health concluded that “in the context of the levels studied, data do not suggest a protective association between higher Se intake or status in relation to prostate or lung cancers, and *data are insufficient to establish whether or not selenium is associated with the risk of developing breast or colorectal cancers* [33]”. The 2014 update of the Cochrane review on Se and cancer prevention came to similar conclusions [34]. That being said, such reviews rely heavily on the results of two major trials described below, conducted in the American population with largely adequate baseline Se status.

The strongest evidence of a protective effect of Se against CRC comes from the Nutritional Prevention of Cancer (NPC) trial—a double-blind, placebo-controlled randomized trial of treatment with 200 μg Se per day in 1312 subjects from south-eastern USA with a history of non-melanoma skin cancer, which was also the primary outcome [35]. After a mean follow-up of 4.5 years, there was no effect of Se on the primary outcome, but there was a significant 50% reduction in cancer mortality, as well as reduction in cancer incidence of 37% for all cancers, 67% for prostate cancer, 58% for CRC and 46% for lung cancer. However, the follow-up was extended to 7.9 years in a non-blinded fashion, by the end of which period the incidence remained significantly reduced only for total (25%) and prostate (52%) cancers [36], as well as lung cancer in subjects in the bottom tertile of baseline plasma Se (<106 ng/ml) [37].

The results of the largest double-blind, placebo-controlled randomized trial to date (35,533 subjects), the Selenium and Vitamin E Cancer Trial (SELECT), investigating the effect of Se supplementation (200 μg/day) on prostate cancer incidence with CRC as one of the secondary outcomes, have been null [38]. However, the baseline Se status was likely too high for any potential benefit to manifest itself. The median baseline serum Se was 136 ng/ml [38], while in the NPC trial the median baseline plasma Se was 113 ng/ml [36] (plasma and serum Se assays yield similar values). Also, few subjects in the SELECT trial had baseline serum Se below 106 ng/ml, which was the plasma Se range in the NPC trial for which there was the strongest evidence of beneficial effects of Se supplementation on prostate cancer risk. Concurrently, there was no evidence of benefit in the highest baseline Se status category (>123 ng/ml) in the NPC trial [36, 39].

Even before the results of SELECT were available, Rayman proposed that due to the lower Se status of Europeans, Europe was a more suitable geographical area for conducting a primary prevention Se trial the likes of SELECT, as stronger effects would be expected in such a population [26]. Other than the baseline Se status of the study population, another issue which is recommended to be taken into account in study design of future Se trials is the measurement of single nucleotide polymorphisms influencing Se metabolism, which may have contributed to the conflicting findings on Se and cancer risk in humans [40]. Moreover, Se status assessment itself represents a challenge, as relatively little is known about the performance of currently used biomarkers as indices of long-term Se exposure. In a recent pooled analysis of individual participant data from fifteen prospective studies, blood Se concentration (short-term exposure biomarker) was not associated with risk of total prostate cancer, while nail Se concentration (longer-term exposure biomarker) was inversely associated with this risk [41]. These results call for more attention being paid to Se exposure assessment in future studies, as well as for a reappraisal of the Se-prostate cancer relationship.

In principle, the current understanding of the impact of Se on cancer risk, if any in humans, suggests that it is largely mediated via the influence of Se status on the expression of selenoproteins, with different selenoproteins having different saturation plasma/serum Se thresholds [30]. Both observational data in humans [42], as well as basic research on Se [43] suggest the existence of a U-shaped curve—or ‘a split “Dr. Jekyll and Mr. Hyde” personality’ of some selenoproteins, as illustratively described by Hatfield and colleagues [43]—of the Se status or intake and the anti- or pro-tumourigenic effects. Anti-tumourigenic effects of Se have been observed in animal studies utilizing doses of Se an order of magnitude higher than required for saturation of most selenoproteins’ expression [30]. This implies that either non-selenoprotein Se compounds—i.e. low molecular weight Se-metabolites—exert such effects or some under-researched, and possibly even undiscovered, selenoprotein(s) (e.g. those expressed by immune cells) have substantially higher Se requirements for maximum expression.

In the context of observational studies, it is reasonable to assume that revealing a ‘true’ Se-cancer risk association may only be possible, when the range of Se statuses in the study sample encompasses both deficient or suboptimal, as well as optimal concentrations of selenoproteins relevant to a particular type of cancer [44].This may have been the case in the recently published case–control study nested in the whole EPIC cohort on Se status and CRC risk [45]. It found that serum levels of SePP were inversely associated with CRC risk, with the association being more apparent in women, while serum Se was significantly associated with CRC risk only in women, and not in both sexes combined. The finding of the associations being more evident in women is particularly relevant to the EPIC-Oxford cohort, in which women constitute 78% of all subjects (albeit no evidence of effect modification of CRC risk in vegetarians by sex was reported in any of the publications on cancer incidence or mortality in EPIC-Oxford [9–12]).

## Taking a step back—what is actually “selenium”?

It should be emphasized that unlike many other minerals of nutritional relevance, dietary Se is not a discrete substance, but rather a group of compounds containing Se [46], which have different metabolic pathways [47], as well as half-lives and Se bioavailabilities (which are generally high) [48]. Simply drawing attention of a reader with basic knowledge of chemistry to the fact that Se has four oxidation states of -II, 0, IV and VI is likely to be instantaneously and intuitively informative of the biochemical diversity and complexity to be expected. Briefly, Se is present in foods mostly as inorganic Se (selenate, selenite), selenomethionine (SeMet), methylselenocysteine (MeSeCys) and selenocysteine (SeCyS) [46–50].

Selenite and SeMet are generally the predominant forms in plants commonly consumed by humans, with notable exceptions of the Se-accumulating cruciferous and allium vegetables, in which substantial proportions of total Se comprise of Se-methyl-selenocysteine and ɣ-glutamyl-Se-methyl-selenocysteine [46, 47]. For animal products, speciation of Se depends on the baseline Se status and whether or not the feed is fortified with inorganic Se [46, 47], which is a common practice in some low-Se countries, including the UK—albeit not mandatory and permissible only at a low level [51]. Provision of inorganic Se increases the SeCys content in animal tissues compared to animals eating Se-containing plant foods, which in turn increases the SeMet content of the tissues [46, 47]. It is thus of note that differences in Se speciation in animal products between the UK and high-Se areas of USA can be expected, which may be of relevance to the differences in relative risk of CRC between British and American vegetarians. Se speciation in fish and shellfish varies greatly between species and may include a substantial proportion of inorganic selenium [52]. Although Se content in this food group is generally high—albeit variable—it may be somewhat less available than from other foods [46–48].

Since the metabolic pathways of all Se-containing compounds intersect at a common metabolite, hydrogen selenide (H_2_Se), it is theoretically possible for all these compounds to be equally utilized for selenoprotein expression. However, due to high reactivity of selenides with oxygen and metals, H_2_Se may not be freely available in sufficient quantities as to not be a limiting factor in some of the Se metabolic pathways [50]. Also, there is a major difference between the two predominant dietary Se compounds, SeMet and SeCys, in that the former can be incorporated non-specifically into proteins by replacing methionine, which allows for storing Se in the organism [49]. This may offer a distinct advantage of prolonged maintenance of Se status by a given dose of Se compared to other Se compounds [49]. However, Se from SeMet—when protein-bound—may be less readily available for conversion to SeCys and further Se metabolism [53]. Indeed, it has been recently shown in a randomized clinical trial in healthy men that selenised yeast (a mixture of Se compounds), but not SeMet, was effective in reducing the levels of oxidative stress biomarkers at similar increases in plasma Se in both intervention arms [54]. The characteristics of SeMet outlined above may serve as a yet another alternative and/or partial explanation for the conflicting results of the NPC trial, in which selenised yeast was used, and SELECT, which utilized SeMet [35, 38, 48, 49, 53, 54].

Appreciating the fact not all dietary Se is ‘created equal’ is important for critical interpretation of studies on health effects of Se, as well as the hypothesis presented in the current review.

## Are vegetarians in the UK at increased risk of low selenium status?

A 7-day duplicate diet study in the UK has found the Se content of approximately 50 vegetarian diets to be 28 μg/day [55], which was lower than the estimated 39 μg/day intake of the general population [55, 56]. Data on Se intakes in British vegans estimated from food records suggest insufficient intakes [57], however, unlike for many other nutrients, this is not a reliable measure of Se dietary intake due to large within-food variation of Se content [58]. Se content of vegetarian diets has also been evaluated by means of a duplicate diet study in Belgium [59] and in Sweden [60]. The mean Se content of 24 h duplicates of vegetarian diets from Belgium was only 13 μg, compared to 50 μg in non-vegetarian diets and 34 μg in macrobiotic diets (i.e. largely vegetarian diets, allowing low consumption of animal flesh, particularly fish, which are a good source of Se [61]). A 4-day duplicate vegan diet samples analysis from Sweden revealed daily Se content of only 7.8 μg, compared to the already low 31 μg in a Swedish mixed diet of elderly subjects [62]. Surprisingly, a similar study by the same group regarding lactovegetarian diets found Se content of 68 μg/day in men and 61 μg/day in women [60]. According to the authors: “This […]might partly be explained by the fact that many of the food items […] were imported, and it is possible that these products were grown in areas that are rich in selenium.”

Only one study directly assessing Se status in British vegetarians was identified. Based on the analysis of toenail concentrations of this mineral in the UK, Judd and colleagues have suggested that vegetarians and vegans may be at increased risk of Se deficiency [63]. The 57 vegetarians and vegans under study had mean toenail Se concentrations of 541 ng/g, which was significantly lower than 685 ng/g in the 67 meat-eaters matched for sex and age. Also, these concentrations were significantly lower in vegans (*n* = 34) than in vegetarians (*n* = 23) at 506 vs 591 ng/g. This may have been at least in part due to eight vegetarians who reported occasional consumption of tuna and had mean concentrations of 644 ng/g. This study was published as a short communication only and unfortunately, it was not stated directly whether or not vegetarians and vegans under study dwelled in the same geographical area as non-vegetarian controls, who resided in Norfolk—a relatively high-Se area within the UK [63].

Plasma Se was assayed in selected participants of the whole EPIC cohort in the nested case-control study of Se status and prostate cancer risk, which included few vegetarians from the EPIC-Oxford cohort [64]. Overall, there were 10 vegetarians and fish-eaters (pesco-vegetarians) each, 56 meat-eaters and no vegans in this analysis. Mean plasma Se values were 68 ng/ml in vegetarians and 81 ng/ml in fish-eaters, compared to 77 ng/ml in meat-eaters (unpublished data). The differences between diet groups were not tested for statistical significance due to the secondary nature of the data, and low numbers of vegetarians and fish-eaters. However, they are in agreement with the overall body of evidence showing lower Se status in vegetarians in low-Se areas and suggest that the same pattern may be present in the EPIC-Oxford cohort—which is one of the underlying assumptions for the hypothesis presented in the current review. (Similarly low numbers of vegetarians and fish-eaters from the EPIC-Oxford cohort were included in the EPIC-nested case-control study of serum Se and SePP, and CRC risk [45] (personal communication with Hughes and Fedirko), also precluding an analysis of Se status by diet group in the EPIC-Oxford cohort).

Available data from other European, low-Se countries consistently [65–72], but not unanimously [73, 74], show lower blood parameters of Se status in vegetarians and vegans compared to non-vegetarians. The results of two studies by Krajcovicová-Kudlácková et al. [73, 74] from Bratislava, Slovakia, which have observed higher Se status in vegetarians than in non-vegetarians, represent the only discrepancy within the studies of Se status in European vegetarians. One of the two studies [74] was published in a non-Medline indexed journal and its full text or abstract is not available. It was only cited in the discussion of the other study [73] which found higher plasma Se in vegetarians, as evidence of previous similar findings (without specifying which Se status biomarkers were used in it). The authors did not provide an in-depth discussion of the possible reasons for the inconsistency of their findings with the rest of the literature. Also, the manuscript was lacking in information on preanalytical sample handling and adequate quality control measures, without which the validity of these data cannot be critically assessed. Similarly, no information was given on methods of recruitment of vegetarians and ascertainment of adherence to vegetarian diet. Should they have been self-reported vegetarians, a substantial proportion of them may have in fact been non-vegetarians [75]. Assuming the validity of the results, one potential explanation for these unexpected findings stems from the Swedish duplicate diet study reporting higher Se content in lactovegetarian diets, which the authors proposed to be due to high intake of foods imported from high-Se areas [61]. Three other subsequent studies by a different group from Bratislava yielded opposite results in regard to plasma Se and other measures of Se status [65–68].

Another ‘special case’ study is an analysis in Finnish vegans and non-vegetarians [72]. It found lower serum Se in vegans; however, the mean values in both groups were noticeably higher than in other European studies. This is because Finland has national legislation which mandates adding Se to fertilizers [76].

The results of these and other identified published studies of Se status in European vegetarians, as well as their Se intakes assessed by duplicate diet analysis, are presented in Table 1. Currently, the most informative and commonly used biomarker of relevance to the anti-tumourigenic Se properties is plasma Se [29], and for the sake of clarity and comparability, the specific values obtained in these studies are presented in Table 1 for this parameter only. For reference, optimal plasma/serum Se is approximately 120 ng/ml and there is no evidence of cancer risk reduction due to increases above this level [29], while levels below 70 ng/ml are considered Se deficiency [58].

The results of two studies on Se status in European vegetarians merit particular attention. First, the 12 month trial assessing trace element status after adopting a lactovegetarian diet from Sweden provided strong evidence that adopting such a diet in the context of a low-Se area decreases Se status, as measured by plasma Se (76 ng/ml at baseline vs ca. 55 ng/ml after 1 year; read from figure) [71]. Second, a cross-sectional study from Germany found no significant differences in extracellular GPx3 activity between vegetarians and omnivores, but serum Se and SePP concentrations were significantly reduced in vegetarians (mean serum Se ca. 74 vs. 93 ng/ml in omnivores; read from figure) [69]—the latter being particularly relevant in the context of CRC [45].

The results of studies of Se status in European vegetarians show that Se status as assessed by plasma or serum is ca. 10–20% lower in vegetarians than in non-vegetarians (but suboptimal in both diet groups), and highlight the considerable contribution of animal products to total Se intake in low-Se areas. However, this is not the case in areas with high soil Se. In contrast to the findings from Europe, North American studies [77, 78], including one study in Adventist vegetarians [78], suggested similar and optimal Se status in vegetarians and omnivores, as assessed by whole blood Se [77], as well as selenium content and GPx activity of milk from vegetarian and non-vegetarian women [78].

Overall, the results of European including some British studies convincingly suggest that lower Se status in British vegetarians than that of meat-eaters is very likely.

## Changes in selenium intakes and status in the UK

A dramatic decrease in the Se status in the UK had been observed over the 1980s in longitudinal studies on same subjects, from mean serum/plasma Se in the range of approximately 110–120 ng/ml in both healthy adults and renal dialysis patients at the beginning of this decade (which were then among the highest in European countries [79]) to as low as ca. 70 ng/ml in Scotland at the beginning of the 1990s [80–82]. A cross-sectional study from late 1980s of a random sample of 1000 adults registered within one general practice in Scotland has observed even lower mean plasma Se of only 60 ng/ml [83].

It should be noted that based on soil and foodstuffs Se content comparison, lower Se status can be expected in Scotland—where 10% of the EPIC-Oxford cohort reside [15]—than in other parts of the UK [84, 76]. However, the differences are likely not to be substantial. A comparison of Se status by UK region in the National Diet and Nutrition Survey (NDNS) 2000–2001 of a representative sample of the UK population found a difference of 5 ng/ml in mean plasma Se between the “London and the South East” region, for which the results were the highest, and Scotland [85].

Only one further study from Scotland assessing Se status was identified for comparability with these findings. It was published in the year 2000, but carried out in an unspecified period, and it found mean plasma Se concentrations of 82 ng/ml [86], which may have been due to differences between the study samples of this and the previous studies [82, 83] or due to an actual increase in Se content in the foodstuffs in the Scottish market. In the 2000–2001 NDNS, the mean plasma Se in Scotland was 86 ng/ml [85]. Given the representativeness of the NDNS samples, i.e. likely including some ill individuals whose Se status can be compromised due to illness, somewhat lower mean Se status can be expected in them than in healthy volunteers, who constituted the samples of most other studies on Se status in the UK discussed in the current review.

The following mean plasma Se concentrations were obtained in this and other editions of the NDNS for the whole UK: 75 ng/ml in non-institutionalized elderly between 1994 and 1995 [87], 87 ng/ml in adults between 2000 and 2001 [85] and 85 ng/ml in both adults and non-institutionalized elderly between 2008 and 2012 [20]. The negligible difference in plasma Se (<1 ng/ml) between the two groups suggests that the results of the 1994–1995 survey in the elderly [87] may have been due to a nadir of Se intake and status in the UK at that time, rather than lower Se status in the elderly than in younger adults. The findings of similar Se status in both groups in the 2008–2012 NDNS [20] are in agreement with other studies, which suggest that apparently healthy elderly subjects have similar [88, 89] or somewhat lower [90] Se status than younger adults.

The decrease in Se status in the UK was due to changes in Se content of food products available in the British market [91], largely resulting from the decline in imports of selenium rich wheat for breadmaking flour from North America [80].

The results of the UK Total Diet Study (TDS, a study of chemical analysis of commonly consumed foods, aiming to estimate mean nutrient contents of a typical diet) are not in full agreement with the findings on Se status in terms of the timing of Se intake/status decrease, suggesting that there was a steady Se content in British diets throughout the 1980s at ca. 60 mcg/day (although there seemed to be a decrease in Se status over this decade), with a sudden decrease (1991–1994) to ca. 40 mcg/day in the 1990s up to early 2000s, and a subsequent increase to 58 mcg/day in the year 2006 [32, 92]. It should be noted that TDS do not provide robust trend data [83] and no changes in food supply between 1991 and 1994 were noted, which could explain the sudden decrease [32]. They were most likely the result of differences in the choice of foods purchased in TDS [32], possibly inflating the actual Se content estimate of the average British diet in the 1991 measurement.

Recent Se status studies from the UK in non-representative, healthy individuals suggest that there may have indeed been a rise in Se content of foods in the British market, reflected in plasma/serum Se concentrations in the 90–100 ng/ml range [93]. Overall, there is strong evidence of a substantial decrease in Se intakes and status throughout 1980s and early 1990s in the UK, and some evidence of a reverse J-shaped curve of Se intakes/status over the last three decades; however, high-quality data to ascertain this are lacking. Moreover, no changes in food supply were identified, which could explain the proposed increases in Se intakes/status over the late 1990s and 2000s.

While the data presented so far are in agreement with the hypothesis proposed in this review, the experience of a nationwide addition of sodium selenite to fertilizers in Finland is the proverbial fly in the ointment. Universal use of fertilizers supplemented with inorganic Se was introduced in this country in 1985 due to very low Se soil content and Se intake [76]. No substantial changes were observed in cardiovascular mortality and cancer incidence trends, including the colon cancer incidence rate, however, as is always the case with such descriptive data, drawing causal inferences is severely limited. It can be argued that either other lifestyle and medical factors were stronger determinants of disease outcomes than the improvement of Se status, or that specifically sodium selenate had no appreciable effect on disease outcomes, which does not automatically rule out the possibility that other Se compounds might have favourably influenced these trends.

## Are the patterns of Se intake associated with the secular trends of CRC risk in British vegetarians?

As presented in Table 2, the patterns of CRC mortality or incidence in British vegetarians followed the presumed patterns of changes in Se intake/status in the UK. First, the HFSS and OVS, which were carried out mostly during the relatively high-Se period in the UK, observed not statistically significant, 15% lower CRC mortality rates in vegetarians than in non-vegetarians [7, 8]. Arguably, the sample sizes in both studies were insufficient to detect small to moderate differences in site-specific cancer rates.

**Table 2:** Temporal changes in Se intakes and Se status in adults, and CRC risk in vegetarians in the UK

Years	Se intake^a^ (μg/day)	Changes in mean plasma/serum Se (ng/ml)	Studies on CRC risk in British vegetarians
1970s			HFSS follow-up begins
1974	60		
1980s	63	Concentrations in the range of ca. 110–120 (among the highest in Europe) begin to decrease	OVS follow-up begins
1985			
1990s	60	Nadir of Se status with the lowest concentrations of 60–70 observed in Scotland (likely slightly lower than in the rest of the UK)	EPIC-Oxford study follow-up begins
1991			
1994	43		
1995	39	UK representative^b^ elderly^c^ 75	
1997	39	Scotland: 82	CRC risk insignificantly lower in vegetarians by 15% in both HFSS and OVS
2000s			
2000	34	UK representative^b^ adults: 87 (from 86 in Scotland to 91 in London and the South East)	
2006	58		EPIC-Oxford CRC RR in vegetarians after 10.7 years mean follow-up: 1.39 (95% CI: 1.01–1.91)
			EPIC-Oxford & OVS combined CRC RR in vegetarians after 12.2 years mean follow-up: 1.12 (95% CI: 0.87–1.44)
2010s		Concentrations in the ca. 90–100 range in healthy individuals, indicating a possible increase in Se status since the 1990s	
		UK representative^b^ adults and elderly^c^: 85 both	EPIC-Oxford & OVS combined CRC RR in vegetarians after 14.9 years mean follow-up: 1.03 (95% CI: 0.84–1.28)

*Se* selenium, *CRC* colorectal cancer, *HFSS* Health Food Shoppers Study, *OVS* Oxford Vegetarian Study

^a^Data from the UK Total Diet Studies (TDS) [33]. Note that TDS do not provide robust trend data— see text for details

^b^Data from the National Diet and Nutrition Survey, carried out in UK representative samples

^c^Non-institutionalized

Second, the EPIC-Oxford study in which increased CRC incidence was observed in vegetarians [9], started soon after the Se intake/status in the UK likely substantially decreased. When data from this point of follow-up in the EPIC-Oxford study were combined with data from the OVS, thus increasing the overall exposure of the study sample to Se, the relative incidence of CRC in vegetarians was 12% higher and no longer significant [10].

After additional follow-up, which took place during the period of possibly higher Se intake/status than the beginning of the EPIC-Oxford study [20, 32, 86, 87, 91, 92], the CRC incidence in vegetarians relative to meat-eaters was virtually the same in the pooled data from the EPIC-Oxford study and OVS [11]. Moreover, the relative CRC incidence rate in vegans, who may be most at risk of low Se intakes in the UK [55], was 1.29 (0.81–2.07), albeit based only on 19 cases [11]. Also, this analysis showed 34% lower CRC incidence in fish-eaters (pesco-vegetarians) than in meat-eaters, with the earlier pooled analysis [10] and EPIC-Oxford study alone [9] showing insignificantly lower CRC incidence in this diet group.

Fish are a rich source of Se [61]; therefore, the lower CRC risk in British fish-eaters than in vegetarians is consistent with the proposed hypothesis of secular changes in Se status/intake modulating CRC risk. However, in the American Adventist Health Study 2, pesco-vegetarians also had significantly lower risk of CRC compared to non-vegetarians, while vegetarians did not [6]. Data on Se status of the cohort are not available and it is difficult to judge it based on the local soil Se concentrations, as the cohort is spread over the USA and Canada. Nevertheless, the Se status of Adventist cohorts is likely to be higher than in EPIC-Oxford, and hence the hypothesized positive impact of Se from fish is likely to be smaller in the USA, if at all existent. It is possible that other nutrients from fish (e.g. vitamin D, omega-3 fatty acids), their synergistic effect (i.e. fish itself), or some other aspect of diet or lifestyle common to American and British fish-eaters/pesco-vegetarians reduces the CRC incidence in these diet groups.

There is some evidence available to support the notion that fish may decrease CRC risk when comparing extreme consumption levels [94], but not when estimating relative risks per 100 g or per times per week consumed [1]. It should be kept in mind that fish-eaters in the EPIC-Oxford study are “fish-allowers,” rather than high fish consumers, with median fish intake of ca. 30 g/day in men and 24 g/day in women, which is similar to that of meat-eaters [95]. 24–30 g of fish translates to up to 21–26 μg of Se in the case of tuna [32], which is substantial compared to both recommended and usual Se intakes in the UK, while not representing a particularly high fish intake.

## Conclusions

**Fig. 1:**
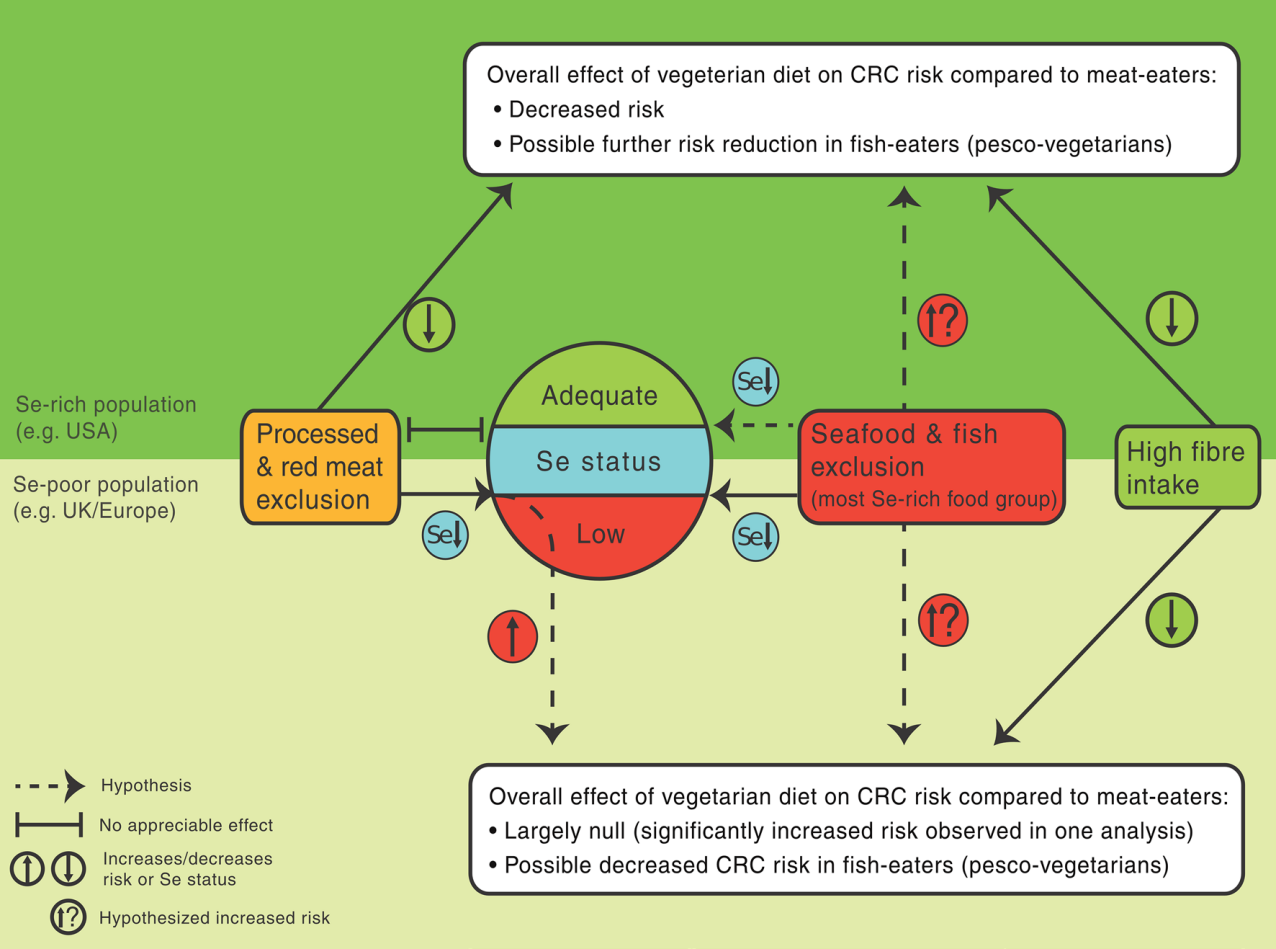
Hypothesized effect modification of the influence of vegetarian diet and its components on colorectal cancer (CRC) risk by selenium (Se) status

Whether or not Se status influences CRC risk in humans is a matter of ongoing debate. Null results in studies of CRC risk in British vegetarians [7, 8, 10, 11] are in disagreement with the established effects of fibre, and processed and red meats on the risk of this type of cancer [1]. Se status represents possibly the most pronounced difference in nutritional status between British (likely low Se status) and American vegetarians (likely adequate Se status), in whom significantly decreased CRC risk has been observed [5, 6]. Figure 1 shows the hypothesized effect modification of the influence of vegetarian diet on CRC risk by high- or low-Se area.

Authors of one of the European studies on Se status in vegetarians went as far as to suggest that “Se supplementation should be recommended to this risk group [vegetarians] of the population” [65]. While undoubtedly Se is a micronutrient of concern in plant-based diets in Se-poor areas, it should be kept in mind that ensuring adequate vitamin B_12_ supplementation (or planning of the diet in regard to fortified foods) is of primary importance both in vegans and considerable proportion of vegetarians. This basic need is often unmet, as reflected by depletion or deficiency rates in vegetarian populations ranging 10–90%—depending on the criterion of deficiency used and life-stage group under study, but regardless of type of vegetarian diet [96]. Thus, a food-based recommendation is desirable and Brazil nuts have been shown to improve Se status [97]. Depending on the soil Se concentration in the country of origin, Brazil nuts may have moderate to high-Se content, ranging from 8 mcg/nut (5 g) in nuts grown in Bolivia, through 18 mcg/nut in Brazil and 33mcg/nut in Peru, to 130mcg/nut when grown in northern countries of South America [98]. Either adequate, country of origin-dependant, “dosing” of Brazil nuts or use of dietary supplements should be exercised by plant-based diet followers in low-Se areas.

Changes in relative risk of CRC in British vegetarians follow the patterns of changes in Se intakes and status in the UK; therefore, the hypothesis that Se status in British vegetarians may influence their CRC risk is worthy of pursuing in future studies. The ongoing EPIC-Oxford study allows for such an opportunity, as blood samples were provided by 19,500 of its participants. A biomarker based study of CRC risk in this population would cover a wide range of intakes, thus making a substantial contribution to the body of evidence on the relationship between Se and CRC.
